# Gout, not hyperuricemia alone, impairs left ventricular diastolic function

**DOI:** 10.1186/s13075-015-0842-8

**Published:** 2015-11-14

**Authors:** Jing-Chi Lin, Chun-Liang Lin, Mien-Cheng Chen, Pey-Jium Chang, Shih-Tai Chang, Chang-Min Chung, Kuo-Li Pan

**Affiliations:** Division of Allergy and Immunology and Rheumatology, Chang Gung Memorial Hospital, Chiayi, Taiwan; Division of Cardiology, Chang Gung Memorial Hospital, Chiayi, Taiwan; Division of Nephrology, Chang Gung Memorial Hospital, Chiayi, Taiwan; Graduate Institute of Clinical Medical Sciences, Chang Gung University, Taoyuan, Taiwan; Division of Cardiology, Kaohsiung Chang Gung Memorial Hospital, Kaohsiung, Taiwan

**Keywords:** Gout, Hyperuricemia, Diastolic dysfunction, Left atrial volume

## Abstract

**Introduction:**

Gout is a common metabolic disorder characterized by hyperuricemia and chronic inflammation. Previous studies show that hyperuricemia accelerates the occurrence and worsening of cardiovascular disease due to LV remodeling. However, it is still unclear whether hyperuricemia is the sole contributor to organic heart remodeling in patients with gout. In addition, there is a paucity of data regarding the association between LV diastolic function and gout. The objective of this study was to investigate the effects of gout on LV diastolic function.

**Methods:**

A total of 173 patients were divided into tertiles based on the following serum uric acid (UA) levels: 1) serum UA ≤ 6.5 mg/dL (n = 54), 2) serum UA >6.5 to ≤8.5 mg/dL (n = 59), and 3) serum UA > 8.5 mg/dL (n = 60).Patients underwent a comprehensive Doppler-echocardiography examination to evaluate LV volume, systolic and diastolic function, and left atrial (LA) volume.

**Results:**

LV diastolic parameters, including diastolic peak early transmitral flow velocity (E), late transmitral flow velocity (A), E/A, peak early diastolic mitral annular velocity (Em), late diastolic annular velocity (Am), Em/Am, E/Em, maximal LA volume index (LAVi) and prevalence of moderate to severe LV diastolic dysfunction were not significantly different between the three groups. Among the population being studied, 108 individuals received a gout diagnosis. Gout patients had greater LV end-systolic dimensions (27.08 ± 4.38 mm, p = 0.006), higher LV mass index (107.18 ± 29.51 g/m^2^, p < 0.001), higher E/Em (10.07 ± 2.91, p = 0.008), and larger maximal LAVi (16.96 ± 7.39 mL/m^2^, p < 0.001) than patients without gout. The prevalence of moderate to severe LV diastolic dysfunction was higher in patients with gout (23 %, p = 0.02).

**Conclusions:**

Gout, not hyperuricemia alone, is associated with LV diastolic dysfunction and LA volume enlargement.

## Introduction

Gout is a common metabolic disorder characterized by hyperuricemia and chronic inflammation [1]. Numerous studies have reported elevated uric acid (UA) as a risk factor for coronary heart disease [2, 3], atrial fibrillation [4] and cardiac mortality [5, 6]. In addition, hyperuricemia is a demonstrated predictor of poor prognosis in various diseases, including acute stroke, congestive heart failure, acute ST-elevation myocardial infarction and chronic renal disease [7–10]. Elevated UA is also associated with left ventricular (LV) hypertrophy in patients without underlying cardiovascular disease [11] and with diastolic dysfunction in patients with heart failure [12]. Hyperuricemia accelerates the occurrence and worsening of cardiovascular disease due to LV remodeling.

In previous studies it has been observed that cardiovascular events are positively associated with gout. Early evidence reported in the Framingham study found a 60 % increased risk of coronary artery disease (CAD) among gout patients [13]. A Taiwanese study demonstrated that the frequency of gout attacks was associated with an odds ratio of 1.18 for myocardial infarction [14]. The MRFIT study found that gout was associated with a 26 % increased risk of acute myocardial infarction [15]. Clinically, the severity of gout, rather than hyperuricemia, is more representative of the chronic inflammation observed in gout patients. Epidemiologically, the study of Kuo et al. demonstrated a link between gout, not hyperuricemia, and a higher risk of death from all causes and cardiovascular diseases [6].

It is still unclear whether hyperuricemia is the sole contributor to organic heart remodeling in patients with gout. Furthermore, there is a paucity of data on the association between LV diastolic function and gout. Therefore, the aim of the current study was to investigate the effects of gout on LV diastolic function.

## Methods

### Subjects and study design

In this prospective study, patients without hyperuricemia, with hyperuricemia, or with gouty arthritis, who underwent echocardiographic examinations, were enrolled between October 2010 and February 2013. Initially, a total of 200 patients were enrolled in this study. Exclusion criteria were the presence of moderate or severe valvular disease, dilated or hypertrophic cardiomyopathy, LV systolic dysfunction (defined as LV ejection fraction (LVEF) <50 %), left bundle branch block, second-degree or third-degree atrioventricular conduction block, permanent cardiac pacemaker implantation, atrial arrhythmias (such as atrial fibrillation, atrial flutter, or atrial tachycardia documented by electrocardiography), congenital heart disease, and a history of previous cardiac surgery. Patients were also excluded if they had poor-qualitied echocardiographic images that precluded analysis.

This study was approved by Chang Gung Medical Foundation Institutional Review Board (IRB 100-3022B) and informed consent was obtained from all patients. The clinical data recorded from the enrolled patients included age, sex, height, weight, systolic blood pressure, serum creatinine levels, serum UA levels, and absence or presence of hypertension, diabetes mellitus, hypercholesterolemia, and smoking. Asymptomatic hyperuricemia was defined as UA ≥7 mg/dL. The diagnosis of gouty arthritis was made according to the American College of Rheumatology (ACR)/Wallace criteria [16]. Patients who had been diagnosed with gouty arthritis using ACR criteria and received medication were also included in the gouty arthritis group. Hypertension was defined as systolic blood pressure ≥140 mmHg and/or diastolic blood pressure ≥90 mmHg. Additionally, patients who were receiving antihypertensive medication were also considered hypertensive. Diabetes mellitus was defined as a fasting plasma glucose level ≥126 mg/dL. Patients receiving oral anti-glucose drugs or insulin for diabetes mellitus control were also considered to have diabetes mellitus. Hypercholesterolemia was defined as either the use of cholesterol-lowering medication or having a total serum cholesterol ≥250 mg/dL in the absence of cholesterol-lowering medication.

### Echocardiography

All subjects received transthoracic echocardiographic examinations at rest in the left lateral decubitus position using a Philips iE33 ultrasound system and S5-1 broadband phased array transducer. The LA diameter in the end-systolic phase, thickness of the interventricular septum (IVS) and posterior wall (PW) in end-diastolic phase, and LV end-diastolic and end-systolic dimensions were all determined using the M-mode in the parasternal long-axis view according to the recommendations of the American Society of Echocardiography [6]. The left ventricular ejection fraction (LVEF), which means LV systolic function, was calculated using Simpson’s method [6]. The LV mass was estimated by the Devereux formula [17]. Then, the LV mass index was obtained as the LV mass/body surface area (BSA). Mitral inflow velocities were evaluated with a 1- to 2-mm sample volume placed at the mitral valve tip by pulse-wave tissue Doppler imaging (TDI) in the apical four-chamber view. Diastolic peak early (E) and late (A) transmitral flow velocity and the E to A ratio (E/A) were measured. In the apical four-chamber view, the peak early diastolic mitral annular velocity (Em) and peak late diastolic annular velocity (Am) were obtained by pulse-wave TDI at the septal site of the mitral annulus. The E/Em ratio was used as an index of LV filling pressure [18]. LV end-diastolic volume (LVEDV) and LV end-systolic volume (LVESV) were measured in the apical four-chamber and two-chamber views using the biplane modified Simpson’s method. Then, the LVEDV and LVESD indexes were calculated as LVEDV/BSA and LVESV/BSA. Maximal left atrial volume (LAV) was also measured in the apical four-chamber and two-chamber views using the biplane modified Simpson’s method [19–21]. Maximal LAV was measured just before mitral valve opening, and the maximal LAVi was calculated as maximal LAV/BSA. All echocardiographic parameters were measured from an average of three beats. E/A and E/Em are the parameters of LV diastolic dysfunction clinically. According to the value of E/A and E/Em, moderate to severe LV diastolic function was defined as (1) a pseudonormal pattern: 0.75 ≤ E/A ratio <1.5 and E/Em ratio >10 and (2) a restrictive pattern: E/A ratio ≥1.5 and E/Em ratio >10 [22].

### Statistical analysis

Statistical analysis was performed using SPSS 18.0 statistical software (SPSS Inc., Chicago, IL, USA). Continuous data are presented as mean ± standard deviation (SD), and dichotomous data as number and percentage. Comparisons of continuous variables between groups were performed using analysis of variance (ANOVA) or the unpaired two-tailed *t* test. Categorical variables were compared using the chi square (χ^2^) test. Multiple regression analyses were performed to determine the associations between gout and LV diastolic functional parameters, including E/A, Em, Am, E/Em, and maximal LAV. The multivariate model was adjusted for age, sex, UA, creatinine, diabetes mellitus, hypertension, hypercholesterolemia, and smoking. Statistical significance was set at *p* <0.05. In 20 randomly selected subjects, the interobserver and intraobserver variability in E/Em and maximal LAV measurements were assessed by the coefficient of variation, where differences between measurements were expressed as the ratio of the SD to the mean. Interobserver variability was assessed by two independent observers and intraobserver variability by one observer twice within a two-week period. The interobserver variability of E/Em was 3.3 % and that of maximal LAV was 2.2 %. The intraobserver variability of E/Em was 1.8 % and that of maximal LAV was 1.5 %.

## Results

### Baseline characteristics of patients by UA tertile

A total of 27 patients were excluded because of the presence of LV systolic dysfunction (n = 2), hypertrophic cardiomyopathy (n = 2), permanent cardiac pacemaker implantation (n = 2), atrial fibrillation (n = 2), and poor echocardiographic images that precluded analysis (n = 19). Thus, the final study population consisted of 173 patients, who were divided into tertiles by serum level of UA as follows: group 1: serum UA ≤6.5 mg/dL (n = 54); group 2: serum UA >6.5 to ≤8.5 mg/dL (n = 59); and group 3: serum UA >8.5 mg/dL (n = 60).The mean UA levels of these three groups were 5.03 ± 1.07, 7.63 ± 0.52, and 10.05 ± 1.17 mg/dL, respectively (*p* <0.001). The clinical characteristics of the study population are listed in Table 1. There were no significant differences between the three groups in age, gender or creatinine levels, history of diabetes mellitus, hypertension or hypercholesterolemia, or cigarette smoking. Among the echocardiographic parameters, there were no significantly different LV volume parameters between the three groups, including LV end-diastolic dimensions, LV end-systolic dimensions, LVEDV, LVEDV index, LVESV, LVESV index, and LV systolic function. However, patients grouped by tertiles of increasing UA were significantly associated with a graded increase in LV wall thickness, LV mass, and LV mass index. LV diastolic parameters including E, A, E/A, Em, Am, Em/Am, E/Em, maximal LAVi, and prevalence of moderate to severe LV diastolic dysfunction were not significantly different between the three groups.

**Table 1:** Baseline characteristics of patients grouped by tertiles of serum uric acid and gout

Variables	First (<6.5 mg/dL) (n = 54)	Second (>6.5 and <8.5 mg/dL) (n = 59)	Third (>8.5 mg/dL) (n = 60)	*P* value	Normo-uricemia (n = 35)	Hyperuricemia (n = 30)	Gout (n = 108)	*P* value	Non-gout (n = 65)	*P* value
Age (years)	56.86 ± 15.36	55.79 ± 11.68	58.44 ± 13.38	0.56	53.54 ± 15.64	56.96 ± 16.07	58.20 ± 11.76	0.20	55.09 ± 16.17	0.154
Men	42 (78 %)	54 (92 %)	54 (90 %)	0.064	26 (74 %)	27 (90 %)	97 (90 %)	0.06	50 (81 %)	0.079
Body surface area (m^2^)	1.70 ± 0.20	1.83 ± 0.22*	1.81 ± 0.18*	0.002	1.68 ± 0.19	1.77 ± 0.16	1.82 ± 0.20*	0.003	1.72 ± 0.18*	0.004
Uric acid (mg/dL)	5.03 ± 1.07	7.63 ± 0.52*	10.05 ± 1.17*^†^	<0.001	4.96 ± 1.04	8.87 ± 1.42*	8.19 ± 2.04*	<0.001	6.68 ± 2.3**	<0.001
Creatinine (mg/dL)	1.0 ± 0.43	1.19 ± 0.38	1.34 ± 1.4	0.115	0.83 ± 0.19	1.01 ± 0.13	1.34 ± 1.09*	0.006	0.9 ± 0.19**	<0.001
eGFR	81.68 ± 32.80	81.33 ± 34.91	74.53 ± 31.12	0.41	90.14 ± 30.08	80.67 ± 29.21	75.05 ± 34.20	0.059	85.77 ± 29.83**	0.038
Diabetes mellitus	3 (6 %)	2 (3 %)	6 (10 %)	0.322	2 (6 %)	3 (10 %)	6 (6 %)	0.60	5 (8 %)	0.492
Hypertension	11 (20 %)	12 (20 %)	15 (25 %)	0.781	8 (23 %)	8 (27 %)	22 (20 %)	0.78	16 (26 %)	0.362
Hypercholesterolemia	14 (26 %)	20 (34 %)	19 (32 %)	0.641	17 (49 %)	13 (43 %)	45 (42 %)	0.79	17 (27 %)	0.493
Smoking	6 (11 %)	13 (22 %)	6 (10 %)	0.123	5 (14 %)	5 (17 %)	15 (14 %)	0.95	10 (16 %)	0.657
LV dimensions and volume										
IVS (mm)	9.51 ± 1.32	10.65 ± 1.8*	10.45 ± 1.82*	0.001	9.37 ± 1.61	10.27 ± 1.85	10.49 ± 1.66*	0.004	9.79 ± 1.81**	0.017
LVEDd (mm)	46.76 ± 5.94	48.12 ± 4.97	47.79 ± 5.72	0.404	45.80 ± 6.11	47.15 ± 4.47	48.27 ± 5.53	0.06	46.36 ± 5.52**	0.031
PW (mm)	9.85 ± 1.35	11.15 ± 1.52*	10.94 ± 1.86*	<0.001	9.89 ± 1.56	10.64 ± 1.71	10.93 ± 1.64*	0.006	10.21 ± 1.66**	0.007
LVESd (mm)	26.37 ± 4.55	27.05 ± 4.29	27.04 ± 4.36	0.643	25.64 ± 4.77	27.31 ± 3.77	27.08 ± 4.38	'0.19	26.32 ± 4.43	0.255
LV mass (g/m)	159.6 ± 44.48	195.18 ± 48.37*	192.24 ± 56.28*	<0.001	154.11 ± 48.92	177.80 ± 41.77	193.90 ± 52.59	* < 0.001	164.54 ± 47.97**	<0.001
LV mass index (g/m^2^)	93.78 ± 25.09	107.57 ± 28.39*	106.2 ± 30.61*	0.019	91.14 ± 27.03	100.56 ± 24.09	107.18 ± 29.51	*0.01	95.4 ± 26.57**	0.009
LVEDV (mL)	69.33 ± 19.61	77.29 ± 20.37	72.75 ± 20.21	0.11	66.97 ± 20.19	68.80 ± 19.80	76.49 ± 19.81*	0.02	67.73 ± 20.36**	0.008
LVEDV index (mL/m^2^)	40.83 ± 11.46	42.29 ± 10.81	40.11 ± 10.29	0.538	39.55 ± 10.33	38.51 ± 9.87	42.28 ± 11.12	0.15	39.09 ± 10.3	0.066
LVESV (mL)	19.59 ± 8.14	22.12 ± 8.55	20.07 ± 7.48	0.205	18.54 ± 7.28	19.67 ± 9.25	21.56 ± 7.91	0.12	19.26 ± 8.35	0.105
LVESV index (mL/m^2^)	11.62 ± 5.19	12.1 ± 4.76	11.07 ± 4.03	0.404	10.96 ± 3.91	10.93 ± 4.84	11.98 ± 4.82	0.37	11.08 ± 4.4	0.265
LV systolic function										
LVEF (%)	73.99 ± 9.2	74.28 ± 7.22	73.82 ± 7.26	0.95	72.57 ± 5.42	72.50 ± 6.98	71.95 ± 6.08	0.83	73.67 ± 8.84	0.671
LV diastolic function										
E (m/s)	0.71 ± 0.15	0.66 ± 0.13	0.67 ± 0.17	0.117	0.78 ± 0.15	0.65 ± 0.14	0.67 ± 0.15	0.31	0.69 ± 0.15	0.475
A (m/s)	0.74 ± 0.2	0.73 ± 0.2	0.75 ± 0.19	0.872	0.71 ± 0.17	0.75 ± 0.22	0.74 ± 0.19	0.74	0.74 ± 0.2	0.853
E/A	1.03 ± 0.35	0.99 ± 0.36	0.95 ± 0.34	0.514	1.05 ± 0.38	0.92 ± 0.30	0.98 ± 0.34	0.32	0.99 ± 0.36	0.895
Em (cm/s)	8.12 ± 2.82	7.34 ± 2.22	7.24 ± 2.04	0.102	8.79 ± 3.04	7.94 ± 2.15	7.03 ± 2.02*	<0.001	8.45 ± 2.7**	0.001
Am (cm/s)	8.42 ± 1.57	8.93 ± 1.74	9.14 ± 2.2	0.117	8.24 ± 1.54	9.01 ± 2.14	8.98 ± 1.87	0.11	8.69 ± 1.88	0.414
Em/Am	1.02 ± 0.5	0.86 ± 0.38	0.85 ± 0.34	0.05	1.14 ± 0.57	0.94 ± 0.40	0.82 ± 0.31*	<0.001	1.05 ± 0.52**	0.001
E/Em	9.49 ± 3.05	9.47 ± 2.48	9.67 ± 3.12	0.918	8.58 ± 2.07	8.79 ± 3.31	10.07 ± 2.91*	0.008	8.72 ± 2.74**	0.004
Maximal LAVi (mL/m^2^)	12.94 ± 5.80	16.45 ± 6.59*	15.77 ± 7.18	0.013	11.49 ± 3.36	12.71 ± 4.20	16.96 ± 7.39*f	<0.001	20.34 ± 4.86**	<0.001
Moderate-to-severe LV diastolic dysfunction	7 (13 %)	9 (15 %)	14 (23 %)	0.301	2 (6 %)	3 (10 %)	25 (23 %)	0.02	5(8 %)**	0.016

Data are expressed as mean ± standard deviation or number (percentage). *P <0.05 versus group with uric acid (UA) ≤6.5 mg/dL group or normo-uricemic group. **P <0.05 compared with gout group. ^†^ *P* <0.05 versus group with UA >6.5 and ≤8.5 mg/Dl or the hyperuricemic group. Moderate-to-severe LV diastolic dysfunction: (1) pseudonormal pattern: 0.75 ≤ E/A ratio <1.5 and E/Em ratio >10 and (2) restrictive pattern: E/A ratio ≥1.5 and E/Em ratio >10. *eGFR* estimated glomerular filtration rate, *A* late diastolic peak transmitral flow velocity, *Am* peak late diastolic annular velocity, *E* early diastolic peak transmitral flow velocity, *Em* peak early diastolic mitral annular velocity, *IVS* interventricular septum, *LAVi* left atrial volume index, *LV* left ventricle, *LVEDd* LV end-diastolic diameter, *LVEDV* LV end-diastolic volume, *LVEF* LV ejection fraction, *LVESd* LV end-systolic diameter, *LVESV* LV end-systolic volume, *PW* posterior wall thickness

### LV organic remodeling and diastolic dysfunction in patients with gout

As there were no obvious findings in relation to uric-acid-based grouping, we studied our patients according to diagnosis of gouty arthritis, asymptomatic hyperuricemia and normo-uricemia. Among the study population, 108 individuals received a diagnosis of gout: 65 patients (60 %) received uricostatic agents, 15 (14 %) received uricosuric agents and 77 (71 %) received colchicines. Table 1 shows the baseline characteristics of individuals with and without gout. There were no significant differences in age or gender, history of diabetes mellitus, hypertension or hypercholesterolemia, or cigarette smoking between the patients with and without gout. The patients with gout had significantly higher serum UA levels than those without gout (8.19 ± 2.04 mg/dL, *p* <0.001). Furthermore, patients with gout had significantly worse renal function than those without gout (creatinine 1.34 ± 1.09 mg/dL, *p* <0.001 and estimated glomerular filtration rate (eGFR) 75.05 ± 34.2 ml/min, *p* = 0.059).

The echocardiographic parameters of patients with and without gout are summarized in Table 1. Patients with gout had a significantly thicker IVS (10.49 ± 1.66 mm, *p* = 0.004) and PW than those without gout (10.93 ± 1.64 mm, *p* = 0.006). The LV mass was significantly larger in patients with gout than in those without (193.90 ± 52.59 vs. 164.54 ± 47.97 g/m^2^, *p* <0.001). The LV mass index in the patients with gout was also significantly higher than in those without gout (107.18 ± 29.51 vs. 95.4 ± 26.57 g/m^2^, *p* = 0.009). Additionally, patients with gout had greater LV end-diastolic dimensions (LVEDd) than patients without gout (48.27 ± 5.53 vs 46.36 ± 5.52 mm, *p* = 0.031). There were no significant differences in LV end-systolic dimensions (LVESd), LVEDV index, and LVESV index between the patients with and without gout. Additionally, there were no significant differences in LV systolic function between patients with and without gout. Among the LV diastolic functional parameters, the E, A, and E/A ratio were not significantly different between patients with and without gout. The patients with gout had significantly lower Em than those without gout (7.03 ± 2.02 vs. 8.45 ± 2.7, *p* = 0.001). The Am was not significantly different between groups. The Em/Am ratio was lower in patients with gout (0.82 ± 0.31, *p* <0.001), and the E/Em ratio was higher in the patients with gout (10.07 ± 2.91, *p* = 0.008). Furthermore, the maximal LAVi of the patients with gout was significantly larger than that of the patients without gout (16.96 ± 7.39 mL/m^2^, *p* <0.001). Finally, the prevalence of moderate to severe LV diastolic dysfunction was higher in patients with gout than in patients without gout (23 % vs. 8 %, *p* = 0.016). There were significant differences between the normo-uricemia and hyperuricemia group, in uric acid, creatinine (0.83 ± 0.19 vs. 1.01 ± 0.13 mg/dL, *p* <0.001), IVS (9.37 ± 1.61 vs. 10.27 ± 1.85 mm, *p* = 0.41) and LV mass (154.11 ± 48.92 vs. 177.80 ± 41.77 g/m, *p* = 0.42). There were no further statistically significant differences between the normo-uricemia and hyperuricemia groups (data not shown).

On multivariate analysis, the associations between gout and LV diastolic functional parameters, including Em, Am, Em/Am, E/Em and maximal LAVi, were significant when controlled for age, sex, UA, creatinine, diabetes mellitus, hypertension, hypercholesterolemia and smoking in the study groups (Table 2).

**Table 2:** Multivariate regression analysis of the association between left ventricular diastolic functional parameters and the gout study groups (n = 173)

Variable	Unadjusted^a^		Model 1^b^	
	*ß*	*P*	*ß*	*p*
E/A				
Gout	−0.01	0.894	0.077	0.237
Em (cm/s)				
Gout	−0.282	<0.001	−0.208	0.002
Am (cm/s)				
Gout	0.062	0.414	−0.019	0.815
Em/Am				
Gout	−0.257	0.001	−0.163	0.016
E/Em				
Gout	0.214	0.005	0.172	0.02
Maximal LAVi (mL/m^2^)				
Gout	0.35	<0.001	0.294	<0.001

^a^Including gout. ^b^Adjusted for age, sex, uric acid, creatinine, diabetes mellitus, hypertension, hyperlipidemia, and smoking. *A* late diastolic peak transmitral flow velocity, *Am* peak late diastolic annular velocity, *E* early diastolic peak transmitral flow velocity, *Em* peak early diastolic mitral annular velocity, *LAVi* left atrial volume index

## Discussion

The major finding of this study was that gout impacts LV diastolic dysfunction and LA volume enlargement. LV diastolic remodeling may be a predictor of adverse cardiac events in gout patients. LV diastolic dysfunction refers to an abnormality of diastolic distensibility, filling, or relaxation of the LV [23]. LV diastolic dysfunction has major effects on the LA, which is a transporting chamber that receives blood from the pulmonary veins and conveys it to the LV through both passive and active diastolic filling [24, 25]. Echocardiographic evidence of diastolic dysfunction is an independent risk factor for heart failure [26]. LV diastolic dysfunction increases atrial afterload, atrial stretch, and atrial wall stress as a result of dilation, which can promote atrial fibrillation. In the study of Tsang et al., more severe diastolic dysfunction and increased LAVi were associated with an increased risk of incident atrial fibrillation [27]. In addition, diabetes mellitus patients with moderate to severe diastolic dysfunction have an increased risk of incident atrial fibrillation [28]. The severity of LV diastolic dysfunction and LA enlargement can predict cardiovascular events.

Elevated serum UA levels are associated with diastolic dysfunction in chronic heart failure patients with LVEF <45 %, due to free radical-mediated endothelial damage resulting from increased xanthine oxidase activity [12]. High UA levels were associated with increased LV mass, end-diastolic LV dimensions, and IVS thickness compared with patients with low UA levels in the present study (Table 1) and that of Krishnan et al. [29]. A possible mechanism to explain this observed relationship is the accumulation of reactive oxygen species due to upregulation of xanthine oxidase in hyperuricemia.

Gout is a metabolic disorder characterized by hyperuricemia and a type of chronic inflammatory arthritis induced by deposition of monosodium urate crystals in the synovial fluid and other tissues [1]. Accumulating evidence from previous studies supports the existence of a repetitive and progressive state of inflammatory activation that is strongly associated with the progression of ventricular diastolic dysfunction and characterized by the intense release and activation of circulating cytokines [30, 31]. Whether hyperuricemia alone contributes to LV organic and functional remodeling in gout patients remains contentious. The current study is the first to evaluate LV diastolic function in gout patients and to determine the role of chronic gout-related inflammation in LV diastolic dysfunction remodeling. In the present study, LV diastolic functional parameters, including E/A, Em, Am, E/Em, and maximal LAVi, were not significantly different between the groups with low and high serum UA. Furthermore, there was no association between serum UA and moderate-to-severe LV diastolic dysfunction (Table 1).

**Fig. 1:**
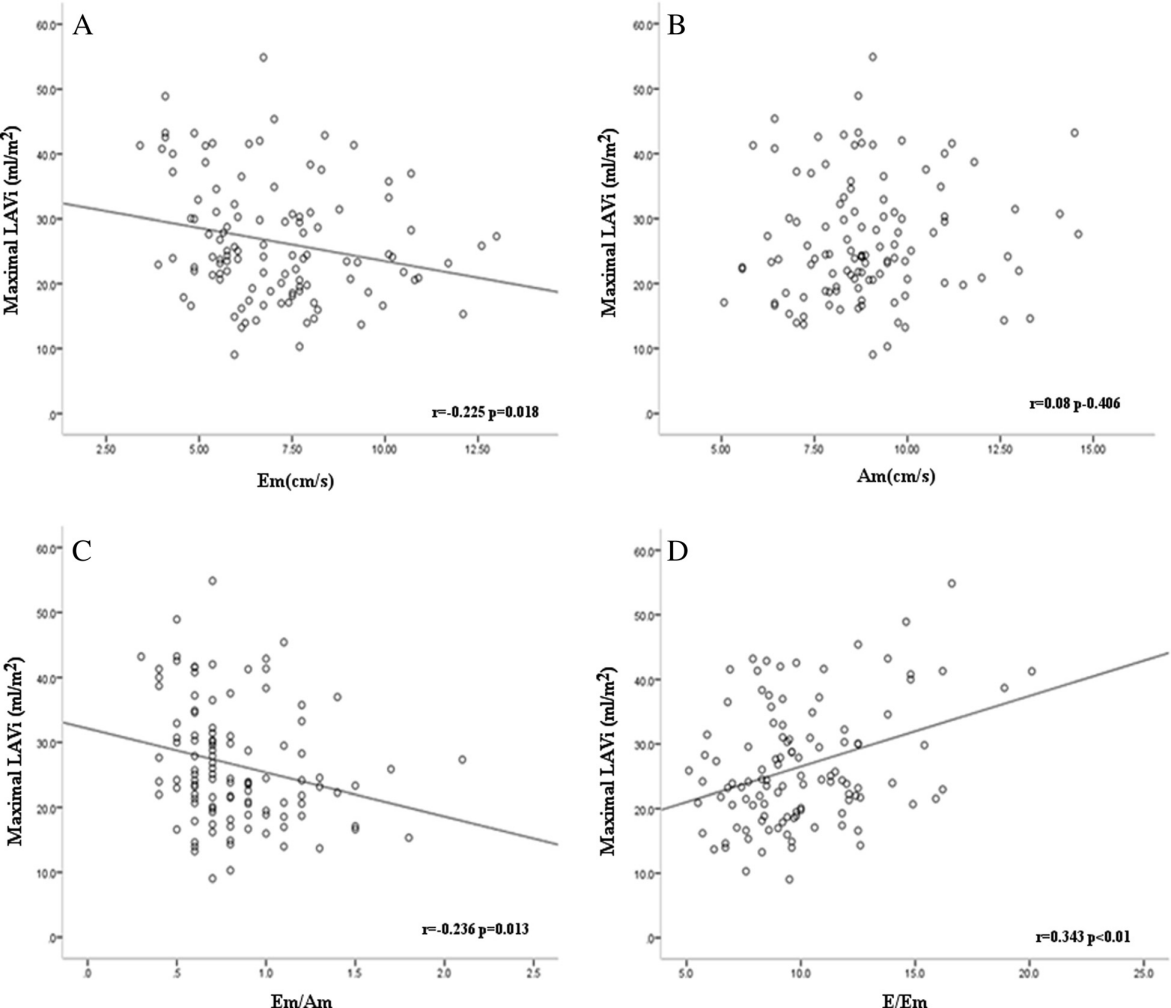
Linear regression analysis and Pearson correlation coefficients in patients diagnosed with gout (n = 108), for association between maximal left atrial volume index (*LAVi*) and peak early diastolic mitral annular velocity (*Em*) (**a**), peak late diastolic annular velocity (*Am*) (**b**), Em/Am (**c**), and early diastolic peak transmitral flow velocity (*E*)/Em (**d**)

Interestingly, lower Em and higher E/Em were detected in patients with gout than in those without gout, and the prevalence of moderate-to-severe LV diastolic dysfunction was higher in patients with gout than those without gout (23 %, vs. 8 %, p = 0.016; Table 1); however, there was no difference between those with normo-uricemia and hyperuricemia. E/Em is an excellent predictor of LV filling pressure and a parameter of LV diastolic function [32, 33]. A possible explanation for these observations is that patients with gout present with monosodium urate crystal-mediated inflammatory cytokines including tumor necrosis factor-α (TNF-α) and interleukin-1β (IL-1β), which have been implicated in the pathogenesis of myocardial dysfunction [34]. Maximal LAVi was also larger in patients with gout than those without gout and there was no difference between the normo-uricemia and hyperuricemia group. LA enlargement is primarily the result of pressure or volume overload, and can serve as an index of atrial remodeling [35]. In our study gout was an independent factor of impaired LV diastolic dysfunction, including decreased Em, decreased Em/Am, and increased E/Em (Table 2). Maximal LAVi was negatively associated with Em (*r* = −0.225, *p* = 0.018) and positively associated with E/Em (*r* = 0.343, *p* <0.001) (Fig. 1). Gout impairs LV compliance, which increases LV filling pressure. During ventricular diastole, the LA is directly exposed to LV pressure through the open mitral valve. Consequently, LA pressure increases in order to maintain adequate LV filling, which results in increased LA wall tension and ultimately LA enlargement. Therefore, enlarged LA volume may reflect the severity of diastolic dysfunction in gouty arthritis patients. These results suggest that in patients with gout, LV diastolic remodeling might be associated with the inflammation mediated by monosodium urate crystals rather than isolated hyperuricemia.

There are several limitations in the present study. First, further follow-up cohort studies are necessary to demonstrate whether LV diastolic remodeling is predictive of adverse cardiac events in patients with gout. Second, the biological link between gout and LV diastolic dysfunction remains unclear; our current hypotheses and results require further clarification through additional studies.

## Conclusion

Gout, rather than hyperuricemia, is associated with LV diastolic dysfunction and LA volume enlargement. LV diastolic remodeling may be a predictor of adverse cardiac events in patients with gout.

## Abbreviations

BSA: body surface area
IVS: interventricular septum
LV: left ventricular
LVEF: left ventricular ejection fraction
LVEDV: left ventricular end-diastolic volume
LVESV: left ventricular end-systolic volume
LA: left atrial
LAVi: left atrial volume index
LAV: lLeft atrial volume
PW: posterior wall
TDI: tissue Doppler imaging
UA: uric acid
